# Supplementary material for: Probiotics Supplementation Attenuates Inflammation and Oxidative Stress Induced by Chronic Sleep Restriction
Source: Nutrients. 2023 Mar 21;15(6):1518. doi: 10.3390/nu15061518 (PMC10054086; doi:10.3390/nu15061518)
Supplement: Supplementary file 1 [file nutrients-15-01518-s001.zip › nutrients-2274199-supplementary.pdf]

Table S1. List of used primary and secondary antibodies.

	Company	Code	Dilution
Anti-Nitrotyrosine antibody [EM-30]	AbCam	ab125106	1:1000
Anti-Dityrosine antibody [10A6]	AbCam	ab243067	1:2000
Anti-4 Hydroxynonenal antibody [12F7]	AbCam	ab243070	1:2000
Anti-Ogg1 antibody	Santa Cruz B.	Sc-376935	1:500
8-oxo-2'-deoxyguanosine (8oxodG)	Santa Cruz B.	Sc-66036	1:500
ionized calcium-binding adapter molecule 1 (Iba1)	AbCam	ab178846	1:2000
ionized calcium-binding adapter molecule 1 (Iba1)	Wako	019-19741	1:500
Goat anti-Rabbit IgG (H+L) Alexa Fluor Plus 488	Thermo Fisher	A32731	1:600
Recombinant Anti-IL-6 antibody [EPR23819-11]	AbCam	ab259341	1:1000
Anti-IL-10 antibody	AbCam	ab9969	1:1000
Recombinant Anti-TNF alpha antibody [EPR22598-212]	AbCam	ab255275	1:1000
Recombinant Anti-IL-1 beta antibody [EPR23851-127]	AbCam	ab254360	1:1000
Anti glyceraldehyde 3-phosphate dehydrogenase (GAPDH) (6C5)	Santa Cruz B.	Sc-32233	1:500
Goat Anti-Rabbit IgG H&L (HRP)	AbCam	ab97051	1:5000
Goat Anti-Mouse IgG H&L (HRP)	AbCam	ab205719	1:3000
